# Tantalum Cementless Versus Cemented Total Knee Arthroplasty: A Meta-analysis of Level 1 Studies

**DOI:** 10.5435/JAAOSGlobal-D-22-00219

**Published:** 2023-04-07

**Authors:** Kranti V. Peddada, Connor M. Delman, Christopher T. Holland, John P. Meehan, Zachary C. Lum

**Affiliations:** From the Department of Orthopaedic Surgery, Davis Medical Center, University of California, Sacramento, CA.

## Abstract

Controversy exists on the ideal type of fixation in total knee arthroplasty (TKA). Noncemented fixation has been theorized to improve patient outcomes and longevity of implantation without increased risk of aseptic loosening or radiolucent lines. We sought to compare (1) patient-reported outcomes, (2) survivorship, and (3) revision rates for all-cause and aseptic loosening in a noncemented tantalum total knee with its cemented counterpart.

**Methods:**

Preferred Reporting Items for Systematic Review and Meta-Analyses guidelines were searched using a combination of keywords “trabecular metal,” “tantalum knee,” “total knee arthroplasty,” and “cementless trabecular.” Patient demographics such as age, sex, and body mass index were collected. Outcomes such as Knee Society Scores (KSSs), revisions, and radiolucent lines were recorded for analysis.

**Results:**

Four randomized controlled trials involving 507 patients with an average 5-year follow-up were eligible for meta-analysis. No differences were observed in any demographics such as age, sex, body mass index, nor preoperative KSS. Patients in the cemented cohort improved from preoperative KSS 46.4 to postoperative KSS 90.4 while the tantalum cohort improved from 46.4 to 89.3. No statistical difference was observed in postoperative KSS mean difference between groups. Six patients from the tantalum group underwent revision with one patient for aseptic loosening. Twelve patients from the cemented group underwent revision with four patients for aseptic loosening. No statistical difference was observed between rates of revision, aseptic loosening, or radiolucent line development.

**Discussion:**

Patient-reported outcomes improved postoperatively in both groups. No differences were detected between the cemented and noncemented TKAs in patient-reported outcomes, revision rates, or radiolucent line development. Noncemented tantalum fixation seems equivalent to cemented TKA survivorship. Longer term follow-up of these randomized controlled trials may provide a clearer understanding whether a difference exists.

The number of total knee arthroplasty (TKA) procedures conducted in the United States is expected to be 3.5 million by 2030—a 673% increase from 2005.^1^ 55% to 62% of primary and revision TKAs are expected to occur in patients younger than 65 years, with the fastest growth in patients aged 45 to 54 years.^2^ Long-term TKA durability in the younger population is concerning. The lifetime risk of revision in patients aged 50 to 54 years ranges from 20% to 35%, and up to 1/3 of patients 60 years and younger who underwent primary TKA report residual symptoms and limitations 1 to 4 years after surgery.^3,4^ Although cemented TKA has historically been the benchmark with 10 to 15-year survivability exceeding 90%, high aseptic failure rates have been reported in the younger population.^5,6,7,8,9,10,11^

Noncemented TKA offers the potential benefits of durable fixation through biologic ingrowth, bone preservation and subsequent ease of revision, and reduced third body wear from retained cement fragments.^12,13^ These theoretical advantages were not realized in earlier designs. Failures of the metal-backed patellar implant, including fracture and polyethylene dissociation, resulted in up to 57% revision.^14-16^ Radiostereometric analysis and histologic studies of the noncemented tibial implant demonstrated loosening and poor bony ingrowth.^17,18,19,20,21^

Porous tantalum, henceforth referred to as trabecular metal (TM), has emerged as a promising material to address deficiencies of prior noncemented systems. Ghalayini et al^22^ demonstrated no evidence of loosening of the TM tibial implant and a 1% revision rate at 6 years of follow-up. Numerous studies comparing noncemented TM and cemented TKA systems have demonstrated equivalent results in pain and function scores, as well as in rates of revision surgery.^23,24,25,26,27^ The purpose of this study was to further validate the noninferiority of TM TKA implants compared with cemented TKA through a meta-analysis of Level 1 studies.

## Methods

This meta-analysis adhered to the Preferred Reporting Items for Systematic Review and Meta-Analyses 2020 guidelines.

### Search Strategy

A comprehensive literature search was conducted using PubMed, EMBASE, Cochrane Library, and Google databases from inception to June 2020. The keywords “trabecular metal,” “tantalum knee,” “total knee arthroplasty,” and “cementless trabecular” were implemented in the search. Only English language studies were included. Institutional review board approval was not obtained because the study did not require direct contact with patients or patient-identifying medical record review.

### Inclusion and Exclusion Criteria

Two authors independently reviewed the eligibility of articles in this study. Study design needed to be a randomized control trial (RCT) evaluating a noncemented TM tibial implant compared with a cemented tibial implant. Tibial implants in either group could be monoblock or modular, and femoral and patellar implants could be cemented or noncemented. Studies needed to provide baseline demographic data, preoperative and postoperative validated pain and function scores of patients, radiographic analysis of implants, and implant survivorship data. Review articles, editorials, commentaries, study designs not meeting criteria for a RCT, and studies involving revision TKAs were excluded.

### Data Extraction

Relevant data extracted from each study included primary author, year of publication, level of evidence of study, number of months of follow-up, number of patients in each group, demographic information including average age and sex, average body mass index (BMI), preoperative and postoperative Knee Society Score (KSS) relating to pain, number and cause of revisions in each group, and prevalence of radiolucent lines around the tibia identified radiographically.

### Primary and Secondary Outcomes

The primary outcomes of this study were differences in postoperative KSS pain scores and implant survivorship. The secondary outcome was differences in the number of radiolucent lines identified radiographically between groups.

### Quality Assessment and Risk of Bias

Version 2 of the Cochrane risk-of-bias tool for randomized trials (RoB 2) was implemented to assess study quality and risk of bias in the RCTs. Six domains in the risk assessment of bias were evaluated—risk of bias arising from the randomization process, deviations from the intended deviations due to the effect of assignment to intervention, deviations from the intended deviations due to the effect of adhering to intervention, missing outcome data, measurement of the outcome, and selection of the reported result. A risk-of-bias judgment rated as high, low, or some concerns was determined based on a domain-specific algorithm according to the RoB 2 and answers to the signaling questions of each respective domain (yes/probably yes/no/probably no/no information/not applicable). An overall risk of bias for each study was then determined based on the number of low-risk, high-risk, and some concerns grades from each domain.

### Statistical Analysis

Raw data from the included studies in the meta-analysis were converted to weighted averages based on the number of subjects in each group for continuous variables (age, BMI, and KSS). For categorical variables (sex, all-cause revision, aseptic loosening, and presence of radiolucent lines), the counts were pooled in each group to determine a pooled frequency. Continuous and binary random-effects modeling using the DerSimonian-Laird method for continuous and categorial variables, respectively, were used to determine differences in demographic variables, preoperative and postoperative KSSs, revision rates, and the presence of radiolucent lines. When standard deviations were not available, the values were imputed based on sample size and *P* value. Forest plots of relative risk ratio comparing aseptic loosening and overall revision rates and mean differences comparing postoperative KSSs between groups were constructed. A 95% confidence interval that did not contain 1 for a risk ratio, and 0 for a mean difference was considered statistically significant.

## Results

### Study Selection

**Figure 1:**
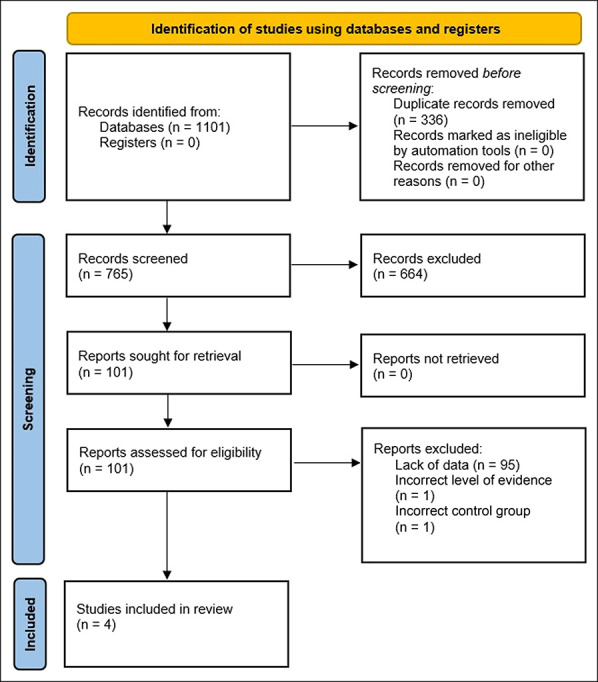
Flowchart depicting the Preferred Reporting Items for Systematic Review and Meta-Analyses 2020 flow diagram for selection of studies in this meta-analysis.

A total of 1,101 potential articles were identified through keyword search in the electronic databases. After removal of 336 duplicates, 765 articles remained for screening. Six hundred sixty-four records were excluded. Abstracts were reviewed for the remaining 101 articles, and 95 were excluded based on the lack of sufficient data. The remaining six articles were appraised in full text. One was excluded for change in study design from an RCT to consecutive series, and one was excluded for not having a cemented tibial implant as a control. Four articles were included in the meta-analysis.^25,26,27,28,29^ Details of the screening process are presented in the Preferred Reporting Items for Systematic Review and Meta-Analyses flowchart in Figure 1.

### Included Studies and Baseline Characteristics

The four included studies were published between 2012 and 2019. The total number of patients was 507, with 248 in the TM group and 259 in the control group. The average follow-up time for all studies was 5 years. Baseline characteristics, including mean age, sex, BMI, and preoperative KSS, were not different between groups. Table 1 summarizes details of the included studies and baseline characteristics of patients. All studies explicitly mentioned that there was no statistical difference between groups.

**Table 1:** Included Studies in Meta-analysis With Demographic and Study Data

Study Primary Author and Year	Average Follow-up (mo)	Sample Size		Mean Age (yrs)		Sex (% Male)		BMI (kg/m^2^)		Preoperative KSS	
		TM	Cemented	TM	Cemented	TM	Cemented	TM	Cemented	TM	Cemented
Fricka 2019	60	41	44	59.8	58.4	36.6	29.5	31.4	31.9	44.3	45.2
Pulido 2015	60	106	126	68.1	68.4	51.9	43.7	31.4	31.8	53.6	53.3
Fernandez-Fairen 2013	60	74	71	61	60	25.7	23.9	29.1	30.5	36.7	33.3
Wilson 2012	60	27	18	60	61	37.0	44.4	32	34	—^a^	—^a^

BMI = body mass index, KSS = Knee Society Score, TM = trabecular metal

a The study by Wilson (2012) used the Western Ontario and McMaster Universities Osteoarthritis Index scores and not KSS.

### Primary and Secondary Outcomes

**Figure 2:**
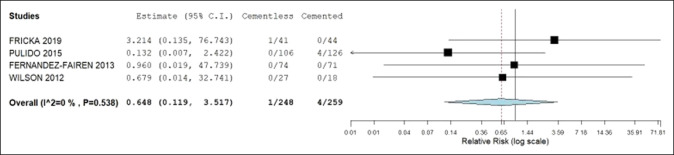
Forest plot demonstrating the relative risk ratio of aseptic loosening between the noncemented and cemented groups. CI = confidence interval

**Figure 3:**
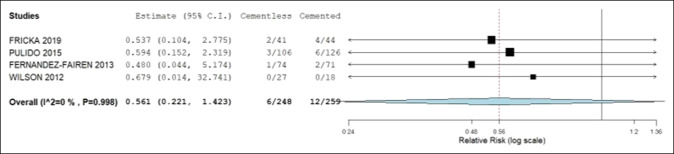
Forest plot demonstrating the relative risk ratio of all-cause revision between the noncemented and cemented groups. CI = confidence interval

**Figure 4:**
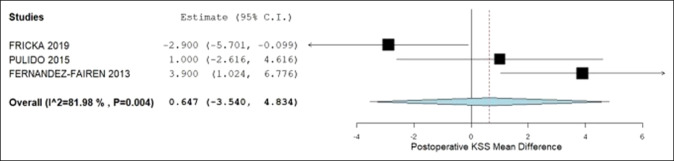
Forest plot demonstrating the mean difference in postoperative KSS between the noncemented and cemented groups. CI = confidence interval, KSS = Knee Society Score

Table 2 presents the pooled analysis of the primary and secondary outcomes. No statistical difference was identified between the cemented and noncemented groups when analyzing the primary and secondary outcomes. The postoperative KSSs was 90.4 and 89.3, all-cause revision rates 2.4% and 4.6%, revision rates due to aseptic loosening 0.3% and 1.4%, and prevalence of radiolucent lines 3.6% and 10% in the noncemented and cemented groups, respectively. The 95% confidence intervals of the mean difference for postoperative KSS and relative risk ratios of revision rates and prevalence of radiolucent lines contained 0 and 1, respectively. Hence, no statistical difference was noted between groups. Figures 2 and 3 illustrate no statistical difference in all-cause revision and aseptic loosening rates within each group. Figure 4 demonstrates that the postoperative KSS score is statistically significantly less in the TM group compared with the cemented group in the study by Fricka and statistically significantly greater in the TM group compared with the cemented group in the study by Fernandez-Fairen. However, the overall meta-analysis model demonstrates no notable difference in postoperative KSSs among the studies.

**Table 2:** Meta-analysis of Clinical and Radiographic Data

Parameter	TM	Cemented	95% CI
Mean preoperative KSS	46.4	46.4	−1.1 to 3.8^a^
Mean postoperative KSS	90.4	89.3	−3.5 to 4.8^a^
All-cause revision (%)	2.4	4.6	0.2 to 1.4^b^
Aseptic loosening rate (%)	0.3	1.4	0.1 to 3.5^b^
Radiolucent lines (%)	3.6	10	0.08 to 5.9^b^

CI = confidence interval, KSS = Knee Society Score, TM = trabecular metal

a Mean difference

b Relative risk ratio

### Quality Assessment and Risk of Bias

Table 3 lists the answers to each question within each domain for the four studies analyzed using the Cochrane RoB 2. All four studies demonstrated a low risk of bias in each domain, yielding an overall low risk of bias for each study.

**Table 3:** Study Quality Assessment Using the Cochrane Risk-of-Bias Assessment Tool

Study	Domain 1	Domain 2a	Domain 2b	Domain 3	Domain 4	Domain 5	Overall risk-of-bias judgment
Fricka 2019	Low	Low	Low	Low	Low	Low	Low
Pulido 2015	Low	Low	Low	Low	Low	Low	Low
Fernandez-Fairen 2013	Low	Low	Low	Low	Low	Low	Low
Wilson 2012	Low	Low	Low	Low	Low	Low	Low

## Discussion

This study sought to determine differences in clinical and radiographic outcomes between tibial TM noncemented and cemented implants. Four RCTs were chosen for this meta-analysis following a methodical screening process from electronic databases. The meta-analyzed data revealed that no statistical differences existed between these groups when comparing postoperative KSSs, all-cause revision rates, and revision rates due to aseptic loosening. The cemented group had a statistically higher prevalence of radiolucent lines at the final follow-up. The bias control analysis demonstrated that there was an overall low risk of bias in the included studies, bolstering the internal and external validity of this meta-analysis.

The theoretical advantages TM tibial implants confer based on in vitro studies are being realized clinically, as demonstrated by this study. The low all-cause revision rate of 2.2% at nearly 5 years is likely a reflection of the superior biological and mechanical properties of porous tantalum compared with earlier noncemented designs. First, the high friction coefficient against bone enhances the initial stability needed before ingrowth.^30^ In fact, the surface coefficient of friction of net-shaped porous tantalum ranges from 0.98 to 1.10, which is approximately twice as high as porous-coated and sintered bead materials.^31^ Second, its high porosity of 75% to 85% facilitates bony ingrowth up to 80%, facilitating longer term survivorship.^20,32,33^

Although Level 1 evidence of TM tibial implants is mostly limited to an average of 5 years of follow-up, several studies suggest that these encouraging results noted are likely sustainable. Niemelainen et al and Hayakawa et al demonstrated a 0% revision rate for loosening in 1143 primary TKAs from the Finnish Arthroplasty Registry at 7 years and 29 primary TKAs at 8 years, respectively.^34,35^ Henricson and Nilsson reported radiostereometric analysis data comparing monoblock TM and cemented NexGen tibial implants. The maximum total point motion and subsidence were statistically higher in the noncemented group at 3 months, likely because of inferior initial stability compared with cemented implants. However, displacement stabilized thereafter with no TM implant demonstrating greater than 0.3-mm change in maximum total point motion between 2 and 10 years.^24^

Although midterm studies suggest equivalent clinical, functional, and radiographic outcomes, long-term data are lacking to support superiority of cemented or TM noncemented implants. One potential advantage of TM implants is that the yield and ultimate strength are 10 times greater than the subchondral bone while the modulus of elasticity is roughly equivalent. This enables support of physiologic loads while potentially minimizing stress-shielding and preserving bone stock.^36-38^ Theoretically, this may increase implant durability and facilitate revision surgeries as bone loss is minimized. However, one concern is that the high porosity of TM increases the intrinsic permeability of tantalum scaffolds to 2.1 × 10^−10^ to 4.8 × 10^−10^ m^2^, possibly facilitating particle-mediated aseptic loosening through wear particle transport to the bone implant interface.^39^ Ultimately, long-term studies are needed to determine whether these mechanical and biological properties bear any clinical relevance.

Strengths of this study include its Level 1 study design being a meta-analysis of RCTs and strict adherence to study selection based on a validated risk assessment of bias tool. One limitation is that none of the included studies could be double-blinded because the surgeons and radiologists interpreting the plain radiographs were aware of the implants used. However, it was thought that the assessment of the outcomes was unlikely to be influenced by knowledge of the intervention because radiologists were blinded to the nature of this study and functional outcomes were ascertained by a third-party blinded to this study. Hence, an overall low risk of bias was still computed according to the RoB 2 algorithm.

Future studies should focus on long-term outcomes comparing TM noncemented and cemented implants, specifically in patients younger than 65 years. Cost differences between implants should also be examined looking at immediate cost and potential savings because of potentially reduced need for future revision surgeries. Clinical and cost differences can also be investigated between total noncemented and hybrid fixation TKAs.

This meta-analysis of RCTs comparing cemented and TM noncemented tibial implants demonstrates equivalent clinical and radiographic outcomes at the midterm follow-up. No statistical difference was noted between groups for postoperative KSS, all-cause revision rates, and aseptic loosening revision rates. Fewer radiolucent lines were observed at the final follow-up in the TM noncemented group. Although porous tantalum seems to offer more durable fixation compared with earlier noncemented designs, additional studies with longer term follow-up are needed to determine whether it is superior to cemented designs.
